# *QuickStats:* Age-Adjusted Death Rates* from Unintentional Falls^†^ Among Adults Aged ≥65 Years, by Sex — National Vital Statistics System, 1999–2016

**DOI:** 10.15585/mmwr.mm6738a9

**Published:** 2018-09-28

**Figure:**
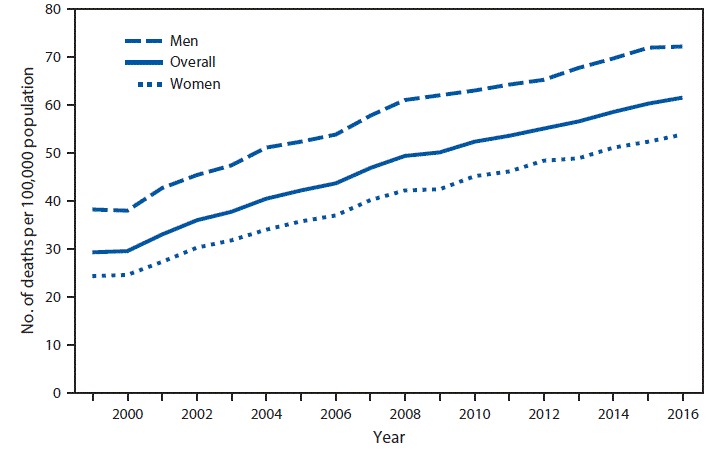
From 1999 to 2016, age-adjusted death rates from unintentional falls among adults aged ≥65 years increased 110% from 29.4 to 61.6 per 100,000. Among men aged ≥65 years, the age-adjusted death rate increased 89% from 38.3 per 100,000 in 1999 to 72.3 in 2016. For women aged ≥65 years, the rate increased 122% from 24.3 per 100,000 in 1999 to 54.0 in 2016. Throughout the period, death rates from unintentional falls were higher for men than women.

**Source**: National Vital Statistics System, 1999–2016. https://www.cdc.gov/nchs/data_access/vitalstatsonline.htm.

For more information on this topic, CDC recommends the following link: https://www.cdc.gov/homeandrecreationalsafety/falls/index.html.

